# Selection and evaluation of appropriate reference genes for RT-qPCR based expression analysis in *Candida tropicalis* following azole treatment

**DOI:** 10.1038/s41598-020-58744-7

**Published:** 2020-02-06

**Authors:** Saikat Paul, Shreya Singh, Arunaloke Chakrabarti, Shivaprakash M. Rudramurthy, Anup K Ghosh

**Affiliations:** Department of Medical Microbiology, Postgraduate Institute of Medical Education and Research (PGIMER), Chandigarh, 160012 India

**Keywords:** Microbiology, Clinical microbiology

## Abstract

*Candida tropicalis* arises as one of the predominant non-*Candida albicans* *Candida* (NCAC) species causing invasive candidiasis in Asian countries. A rise in reports of *C. tropicalis* with a parallel increase in fluconazole resistance has also been observed. The genes and underlying pathways associated with azole antifungal resistance in *C. tropicalis* is still not properly understood. The RT-qPCR is the most promising approach for expression analysis of target genes to understand the mechanisms of resistance. The reliability and reproducibility of this technique depend on the selection of suitable reference genes for the normalization in expression study. The present study investigated the expression stability levels of ten genes including *ACT1*, *EF1*, *GAPDH*, *PGK1*, *RDN5.8*, *RDN18*, *RDN28*, *SDHA*, *TUB1*, and *UBC13* for their suitability in fluconazole treated/untreated *C. tropicalis*. The stability levels of these genes were examined by the ∆∆CT, ΔCT, Pfaffl methods and five independent software including hkgFinder, geNorm, NormFinder, BestKeeper, and RefFinder software. We report, the *EF1* and *ACT1* were the most stable reference genes for normalization and can be used for the gene expression analysis in *C. tropicalis*. To the best of our knowledge, our study is the first to select and validate the reference genes in *C. tropicalis* for RT-qPCR based expression analysis.

## Introduction

*Candida tropicalis*, a non-*Candida albicans Candida* (NCAC) resides in human skin, genitourinary, respiratory, and gastrointestinal tracts as a part of the normal microbiota^1–3^. In Asian countries, *C. tropicalis* has emerged as the predominant NCAC species causing invasive candidiasis (IC), particularly candidemia^4–6^. Fluconazole is the most common antifungal drug used to treat candidemia due to *C. tropicalis*. The rise in IC due to *C. tropicalis* has been paralleled with an increase in fluconazole resistance, especially in Asian countries^4–6^. The differential expression of ergosterol biosynthesis pathway genes, ATP-binding cassette (ABC), and major facilitator superfamily (MFS) drug transporters are directly linked with the azole resistance in *C. tropicalis*^7–9^. Although various studies demonstrated the role of these mechanisms in azole resistant *C. tropicalis*, the principle pathways and regulatory circuits implicated are yet to be elucidated.

Profiling of gene expression is a powerful approach to determine the pattern response to various stimuli including drugs and gives a holistic impression of cellular function in any living cell^10^. Usually, gene expression can be estimated by multiple methods, including RNase protection assay, Northern blotting, real-time quantitative PCR (RT-qPCR), and semi-quantitative reverse-transcription PCR^11^. RT-qPCR has received special attention due to its significantly higher accuracy, sensitivity, and rapidity allowing high throughput results, detection of mRNAs with low-abundance^12^ and mRNA copy number measurement^13^. As a result, RT-qPCR platform has been utilized for diverse applications including gene expression analysis^11,14–16^.

However, the correctness of the results of RT-qPCR depends on numerous technical and biological factors, including the type of samples, method of sample collection, extraction efficiency of RNA, quality and quantity of RNA input, RNA degradation, cDNA synthesis, PCR efficacy, and errors in pipetting^17^. Additionally, the sensitivity, reliability, and reproducibility of RT-qPCR based target gene expression measurement depend on the appropriate normalization^18^. Commonly, normalization is performed by using an internal control gene also recognized as housekeeping gene or reference gene. The selection of inappropriate reference genes for RT-qPCR based expression analysis has produced confusing and unreliable results^19,20^. An appropriate reference gene must be non-regulated, stably expressed, and remain unaltered by experimental and biological conditions^21,22^. However, no gene with all these characteristics has been identified as yet^21^.

The most frequently utilized reference genes for expression analysis, like 18S and 28S ribosomal RNAs, β-actin, tubulin, and glyceraldehyde 3-phosphate dehydrogenase, have presented variable levels of expression under different conditions in diverse cells and tissues, and are consequently inappropriate for the normalization of RT-qPCR^10,17,22–26^. This suggests the necessity to select and validate the appropriate reference genes which are specific for the type of sample and experimental condition used in different studies.

The present study was performed to examine the expression stability of ten candidate reference genes in 20 resistant and 10 susceptible isolates of *C. tropicalis*, collected from different clinical specimens including blood, cerebrospinal fluid, pus, and ascitic fluid. The gene expression of the isolates was evaluated in the presence and absence of fluconazole. We examined the stability of these 10 reference genes by utilizing eight different approaches including, ∆∆CT^27^, ΔCT^28^, Pfaffl^29^ methods and by using 5 different software like hkgFinder^17^, geNorm^21^, NormFinder^30^, BestKeeper^31^, and RefFinder^32^. Furthermore, the selected stable reference genes were validated by analyzing the relative expression levels of different pleiotropic azole resistance genes by using the comparative ∆∆CT method.

## Results

### CT distribution of the reference genes

**Figure 1:**
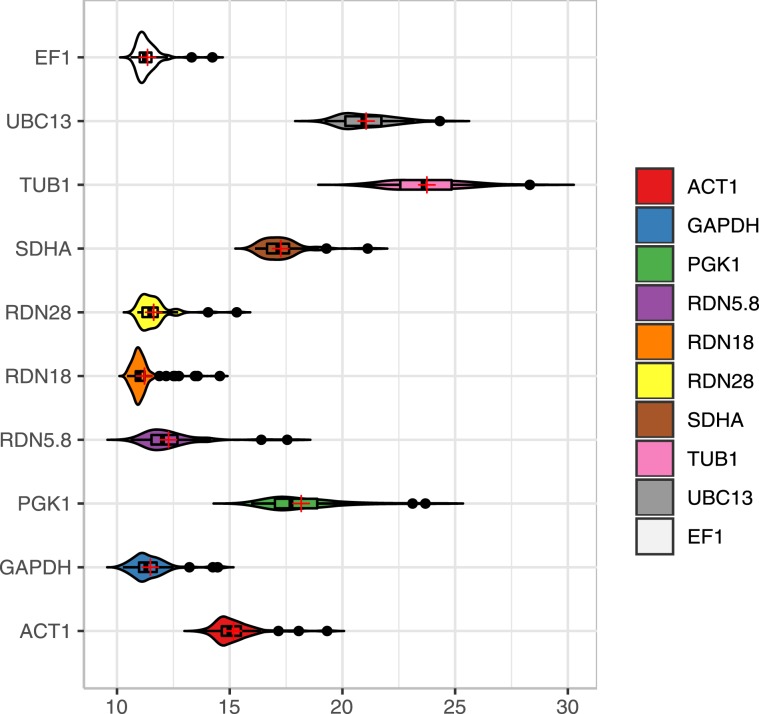
Violin plot representing the distribution of the CT values obtained for 10 candidate reference genes form 60 samples (30 fluconazole treated and 30 untreated control). Violin plot representing minimum value to maximum value with probability density of the data.

Figure 1 representing the CT distributions of 10 candidate reference genes in 60 samples [30 isolates (20 resistant and 10 susceptible)] of *C. tropicalis* in the presence and absence of fluconazole. The instrument generated CT values of the candidate reference genes were ranging from 10.26 to 28.31 (Fig. 1). Four candidate reference genes (*EF1, RDN18, RDN28*, and *GAPDH*) presented significantly lower CT values (p < 0.01), indicating a higher abundance of mRNA transcripts. The CT values of *RDN18* and *RDN28* were uniformly less (~5) in all samples, indicating a higher level of expression. Subsequently, for the stability analysis of *RDN18* and *RDN28*, all the samples (n = 60) were diluted 100 times to increase the CT value up to a detection level (~11). It was difficult to analyse the CT values of *RDN18* and *RDN28* simultaneously with the other reference genes since undiluted samples were used for their analysis. Therefore, *RDN18* and *RDN28*, could not be utilized as reference genes for the expression analysis of target genes.

### Stability analysis of reference genes in *C. tropicalis* following fluconazole stimulation by ΔΔCT, ΔCT and Pfaffl method

#### Stability ranking by ΔΔCT method

To determine the expression stability of the reference genes in fluconazole treated *C. tropicalis*, the CT values were compared between the untreated control (u) and fluconazole treated (t) cells utilizing the formula: average CT Change = CT(u) - CT(t). Two ribosomal RNA subunits *RDN18* and *RDN28*, *EF1*, *SDHA*, *UBC13*, and *GAPDH* were the highly stable genes with CT changes < 0.5. Whereas *ACT1, PGK1*, *RDN5.8*, and *TUB1* were comparatively less stable reference genes with CT changes > 0.5. The stability of RNA expression was validated by comparing with the *EF1*, as it was found to be both suitable and stable. The ΔCT between *EF1* and reference genes was computed by the following formula: [ΔCT(t) = CT(t reference) – CT(t *EF1*) and ΔCT(u) = CT(u reference) – CT(u *EF1*)]. The ΔΔCT(t) was calculated by subtracting the ΔCT of untreated cells from the treated cells. Finally, the levels of reference gene expression in the presence of fluconazole was calculated by transforming the ΔΔCT(t) into 2^−ΔΔCT^ value. The computed ΔΔCT(t) and 2^−ΔΔCT^ results of the 10 candidate reference genes in fluconazole treated samples are provided in Table 1. The 2^−ΔΔCT^ values indicate that *EF1*, *SDHA*, *RDN18*, *RDN28*, *UBC13*, and *GAPDH* were the most stable, while *ACT1, PGK1*, *RDN5.8*, and *TUB1* were comparatively less stable.

**Table 1:** ΔΔCT method based stability analysis of reference genes in *C. tropicalis* treated with fluconazole.

	EF 1	SDHA	RDN 18	RDN 28	UBC 13	GAPDH	ACT 1	PGK 1	RDN 5.8	TUB1
CT Change	0.15	0.18	0.20	0.22	0.27	0.37	0.56	0.69	0.71	0.80
ΔΔCT	0.00	−0.03	−0.05	−0.08	−0.12	−0.23	−0.41	−0.54	−0.56	−0.65
2^−ΔΔCT^	1.00	1.02	1.04	1.06	1.09	1.17	1.33	1.45	1.47	1.57
Ranking	1	2	3	4	5	6	7	8	9	10

#### Analysis by ΔCT method

**Figure 2:**
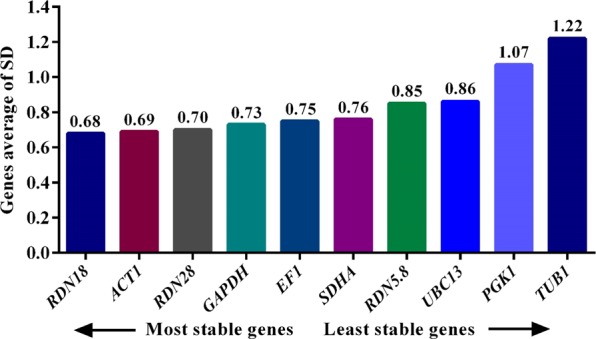
Stability ranking of the 10 reference genes analyzed by ΔCT method.

The stability ranking of the reference genes was also analyzed by the ΔCT method. Among the 10 reference genes examined in this study, *RDN18, ACT1*, and *RDN28* were the most stable reference genes, while *UBC13*, *PGK1*, and *TUB1* were the least stable genes (Fig. 2). However, a difference in the stability ranking of the most stable reference genes selected by ΔΔCT and ΔCT methods was seen.

#### Analysis by Pfaffl method

The expression stability of the reference genes was measured by using the Pfaffl method. According to the ranking order of the reference genes, *EF1, SDHA* and *RDN18* were the most stable, whereas *PGK1, RDN5.8*, and *TUB1* were the least stable reference genes (Table 2). The stability ranking of reference genes by the Pfaffl and ΔΔCT methods were exactly the same and were different from the ΔCT method (Table 3).

**Table 2:** Stability analysis of reference genes calculated by using Pfaffl method.

	EF 1	SDHA	RDN 18	RDN 28	UBC 13	GAPDH	ACT 1	PGK 1	RDN 5.8	TUB1
Average CT controls	11.28	17.17	11.12	11.51	20.92	11.29	14.89	17.83	11.93	23.35
Average CT treated	11.43	17.35	11.32	11.74	21.18	11.66	15.44	18.51	12.64	24.14
Amplification efficiency	94.30	99.70	94.90	97.30	96.50	97.40	98.50	99.30	93.30	95.40
Expression level	1.00	1.03	1.04	1.06	1.09	1.17	1.33	1.46	1.45	1.55
Ranking	1	2	3	4	5	6	7	8	9	10

**Table 3:** Stability ranking of the reference genes by using hkgFinder software.

	EF 1	RDN 28	RDN 18	SDHA	GAPDH	ACT 1	UBC 13	RDN 5.8	PGK 1	TUB1
SD	0.58	0.74	0.78	0.88	0.89	0.92	1.09	1.21	1.6	1.64
log fold change	−0.15	−0.22	−0.2	−0.18	−0.37	−0.56	−0.27	−0.71	−0.69	−0.8
Fold change	1.1	1.2	1.1	1.1	1.3	1.5	1.2	1.6	1.6	1.7
Ranking	1	2	3	4	5	6	7	8	9	10

### Stability ranking of reference genes using five different software

Five independent software including hkgFinder, geNorm, Norm-Finder, BestKeeper, and web-based RefFinder software were utilized to calculate the stability levels of the genes tested. Each software utilizes a considerably different algorithm to assess the stability of the reference genes. Results obtained from these five distinct approaches were used to select the most stable reference genes.

#### hkgFinder analysis

The hkgFinder software selects the most stable reference genes by grading the candidate reference genes with respect to their standard deviation (SD) and fold changes (FC) (Table 4). Out of the 10 candidate genes, the SDs were between 0.58 to 1.64, and the FCs between 1.1 to 1.7. According to hkgFinder, the most stable candidate reference genes identified were *EF1*, *RDN28*, and *RDN28*.

**Table 4:** BestKeeper software base descriptive statistical analysis of reference genes.

	ACT 1	GAPDH	PGK 1	RDN 5.8	RDN 18	RDN 28	SDHA	TUB1	UBC 13	EF 1
N	60	60	60	60	60	60	60	60	60	60
GM (CT)	15.14	11.45	18.11	12.23	11.19	11.60	17.24	23.69	21.02	11.34
AM (CT)	15.17	11.48	18.17	12.28	11.22	11.62	17.26	23.74	21.05	11.35
Min (CT)	13.74	10.26	15.95	10.60	10.45	10.90	16.12	20.88	19.20	10.60
Max (CT)	19.32	14.47	23.68	17.55	14.56	15.31	21.12	28.31	24.32	14.23
SD (±CT)	0.62	0.62	1.23	0.83	0.49	0.47	0.63	1.37	0.90	0.37
CV (% CT)	4.11	5.41	6.76	6.73	4.39	4.04	3.64	5.76	4.26	3.28
Min (x-fold)	−2.64	−2.28	−4.46	−3.09	−1.67	−1.63	−2.17	−7.01	−3.54	−1.67
Max (x-fold)	18.13	8.13	47.64	39.96	10.32	13.05	14.73	24.59	9.83	7.42
SD (±x-fold)	1.54	1.54	2.34	1.77	1.41	1.39	1.55	2.58	1.86	1.29

#### geNorm analysis

**Figure 3:**
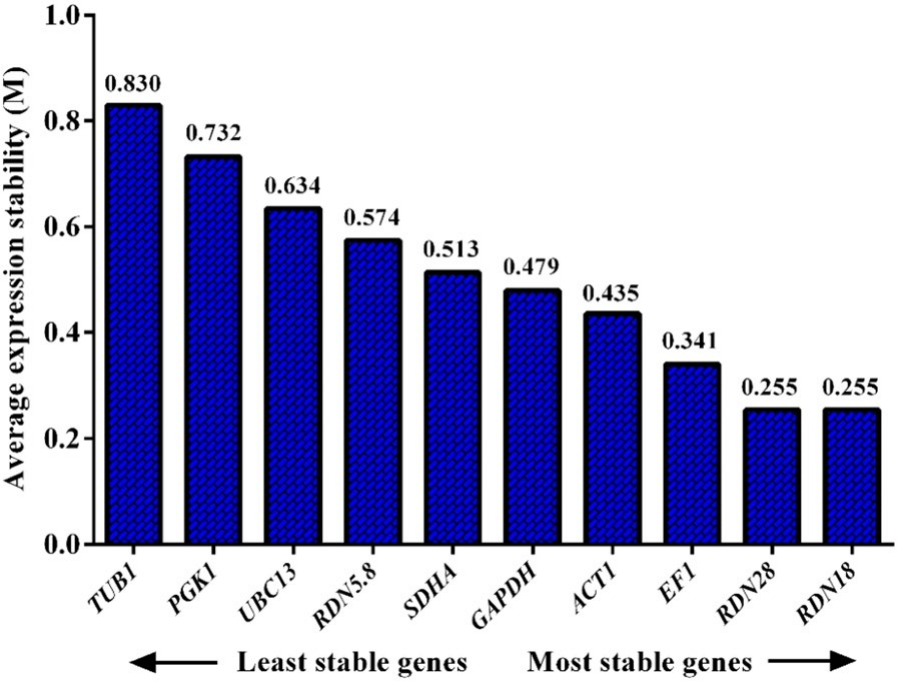
Expression stability values (M) of ten reference genes measured by the geNorm program. Candidate reference genes are ranked from left to right according to their increasing of stability (declining M values).

The geNorm software measures the stability levels of the candidate genes by computing the ‘M’ stability score. A lower an M score indicates higher stability and the default limit of 1.5 is recommended as cut off^21^. All the reference genes had an M score < 1.5 and the geNorm software selected *RDN18, RDN28*, *EF1*, and *ACT1* as the most stable reference genes **(**Fig. 3**)**.

**Figure 4:**
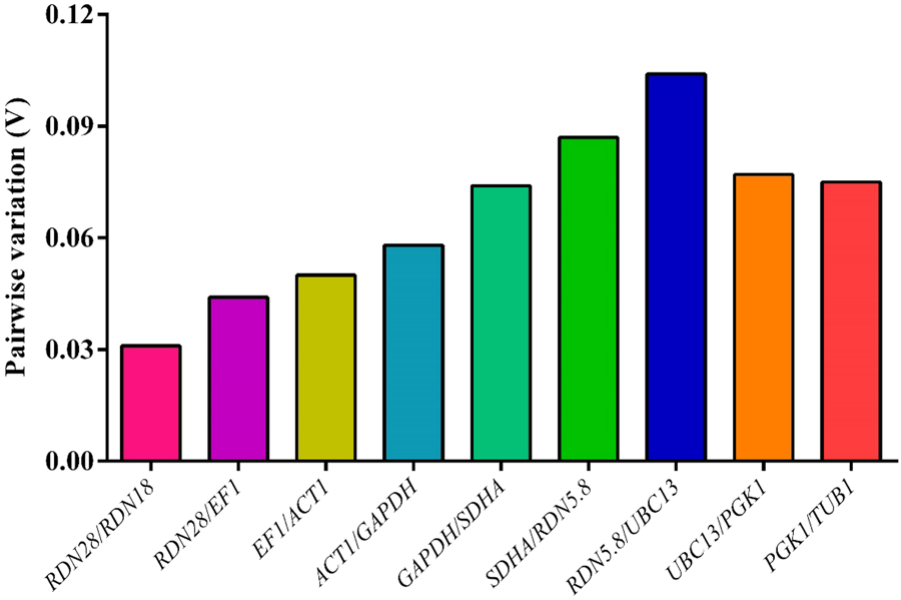
Assessment of the pairwise V values of reference genes determined by geNorm software.

The geNorm software also suggests whether a combination of reference genes is needed or not. Each normalization factor (NF) computed the geometric mean values of two reference genes and calculated their pairwise variability (V value). A combination of reference genes is not recommended at V value < 0.15^21^. In this study, all combinations of genes showed V value < 0.15, indicating no need for combining reference genes for normalization (Fig. 4 and Supplementary Table S4).

#### NormFinder analysis

**Figure 5:**
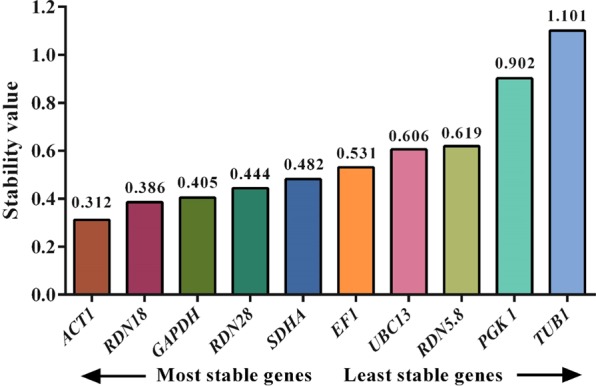
NormFinder based grading of the candidate genes using the stability value.

The NormFinder software analysis grades the reference gene depending upon the stability score, calculated from the intergroup and intragroup expression variability. Although the best reference genes selected by the hkgFinder and geNorm programs were similar (*RDN18*, *RDN28*, and *EF1)* they were considerably different from NormFinder (*ACT1, RDN18*, and *GAPDH)* (Fig. 5 and Table 3).

#### BestKeeper analysis

**Figure 6:**
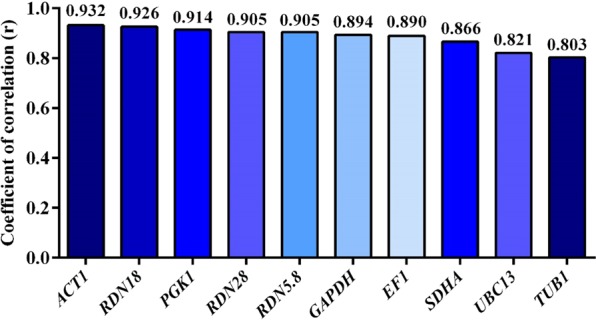
Correlation between the BestKeeper index and the level of reference gene expressions.

BestKeeper program allows for a comparative measurement between different reference genes. The analysis of 10 reference genes exhibited a significantly higher correlation of 0.803 ≤ r ≤ 0.932 among their levels of expressions and the BestKeeper index (r), however, the best correlations were observed for *ACT1, RDN18*, and *PGK1* (Fig. 6).

The BestKeeper software also determined the expression stability by computing both the standard deviation (SD) and as well as the coefficient of variance (CV) of the mean CT values. Out of 10 candidate genes, eight showed SD within the recommended range [0.5 < SD(±CT) ≤ 1.00]^31^. *EF1* showed the lowest SD (0.37) and CV (3.28) indicating higher stability, while *PGK1* and *TUB1* were not found stable as their SD (1.23 and 1.37 respectively) and CV (6.76 and 5.76 respectively) were higher (Table 5). The reference genes (*RDN18, RDN28*, and *EF1*) selected by BestKeeper, geNorm, and hkgFinder were similar while slight discordance with NormFinder was seen (Table 3).

**Table 5:** Ranking of *C. tropicalis* reference gene with respect to expression stability as analysed by six different approaches.

Ranking	2^−ΔΔCT^	ΔCT	Pfaffl	hkgFinder	geNorm	NormFinder	BestKeeper	RefFinder
1	*EF1*	*RDN18*	*EF1*	*EF1*	*RDN18*	*ACT1*	*ACT1*	*RDN18*
2	*SDHA*	*ACT1*	*SDHA*	*RDN28*	*RDN28*	*RDN18*	*RDN18*	*RDN28*
3	*RDN18*	*RDN28*	*RDN18*	*RDN18*	*EF1*	*GAPDH*	*PGK1*	*ACT1*
4	*RDN28*	*GAPDH*	*RDN28*	*SDHA*	*ACT1*	*RDN28*	*RDN28*	*EF1*
5	*UBC13*	*EF1*	*UBC13*	*GAPDH*	*GAPDH*	*SDHA*	*RDN5.8*	*GAPDH*
6	*GAPDH*	*SDHA*	*GAPDH*	*ACT1*	*SDHA*	*EF1*	*EF1*	*SDHA*
7	*ACT1*	*RDN5.8*	*ACT1*	*UBC13*	*RDN5.8*	*UBC13*	*GAPDH*	*RDN5.9*
8	*PGK1*	*UBC13*	*PGK1*	*RDN5.8*	*UBC13*	*RDN5.8*	*SDHA*	*UBC13*
9	*RDN5.8*	*PGK1*	*RDN5.8*	*PGK1*	*PGK1*	*PGK1*	*UBC13*	*PGK1*
10	*TUB1*	*TUB1*	*TUB1*	*TUB1*	*TUB1*	*TUB1*	*TUB1*	*TUB1*

#### RefFinder analysis

**Figure 7:**
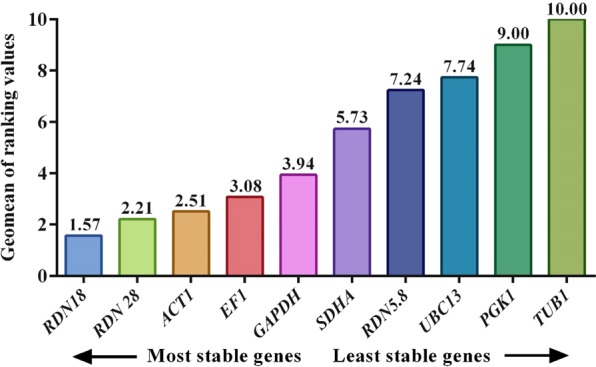
Ranking of reference genes by using the web-based RefFinder tool.

The RefFinder was utilized for the final ranking of the reference genes tested. RefFinder, the web-based tool analyzed the data by integrating NormFinder, BestKeeper, and geNorm for final grading of the reference genes. *RDN18, RDN28, ACT1*, and *EF1* were selected as the best reference genes under every experimental setup (Fig. 7).

Overall *RDN18, RDN28, EF1*, and *ACT1* were the most stable genes. As the amplification efficiency of *RDN18* and *RDN28* was very high, they were excluded. Further, *EF1, ACT1* and the next most stable genes *GAPDH*, and *SDHA* were evaluated. (Table 3).

### Evaluation of selected reference genes

**Figure 8:**
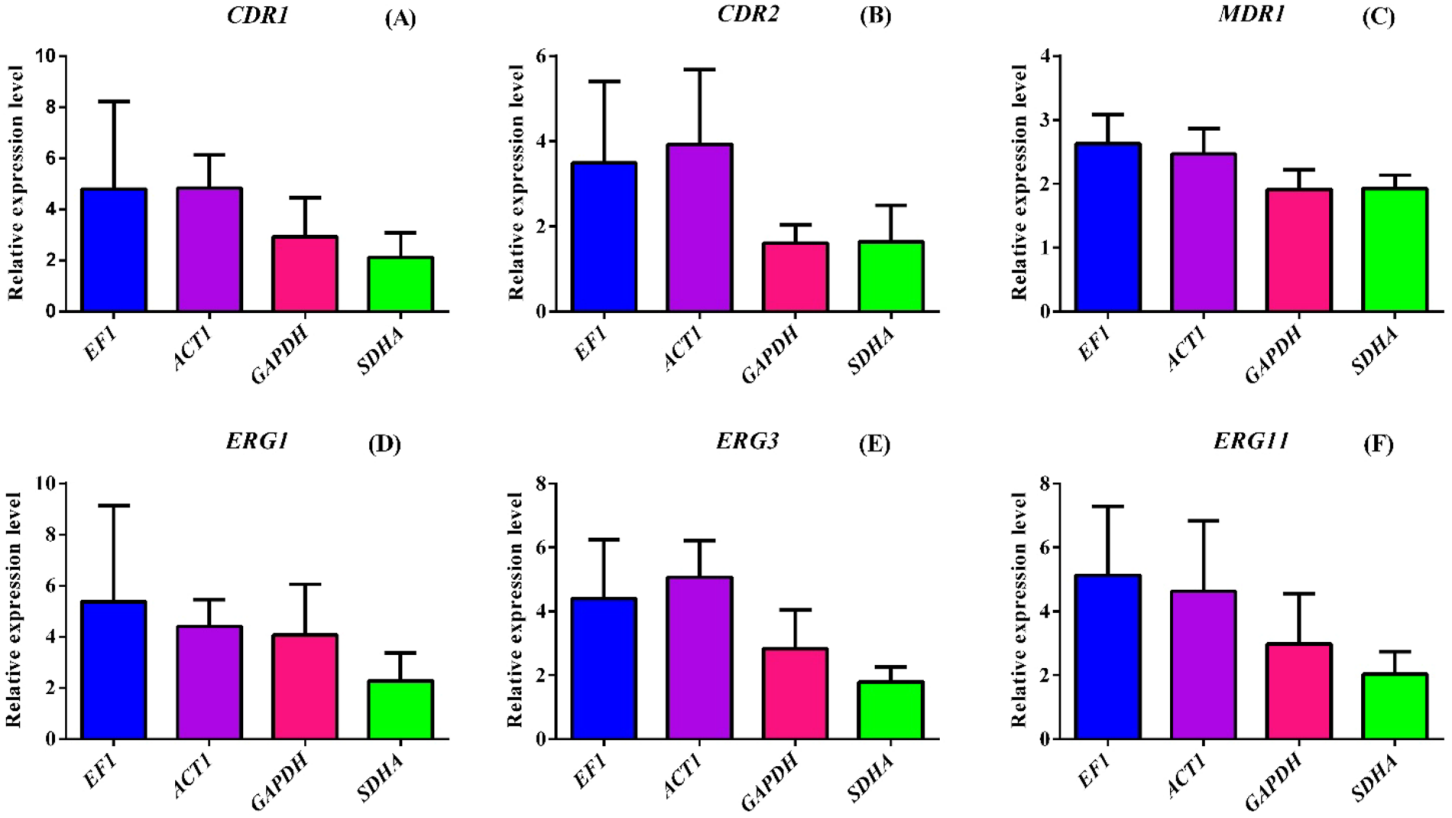
Inducible expression levels of *CDR1, CDR2, MDR1, ERG1, ERG3*, and *ERG11* using *EF1, ACT1, GAPDH*, and *SDHA* as internal controls. To check the statistical significance one way ANOVA with multiple comparisons was performed.

Using *EF1, ACT1, GAPDH*, and *SDHA* as internal control, the inducible expression of azole resistance related genes, *CDR1, CDR2, MDR1, ERG1, ERG3*, and *ERG11* was examined among the resistant isolates^7–9^. The presence of fluconazole, noticeably increased the expression levels of all the genes tested when normalized with *EF1* (2.1 to 9.7 fold) and *ACT1* (2.1 to 7.1 fold). The expression levels of azole resistance related genes were comparatively lower when normalized with *GAPDH* (1.2 to 5.8 fold) and *SDHA* genes (1.1 to 3.3 fold). However, this variation was not significant (p > 0.05) indicating that any of these genes may be utilized for normalization in inducible expression analysis of resistance related genes (Fig. 8).

## Discussion

Appropriate normalization strategies are crucial for the correction of variability in the multistep process of gene expression analysis^10,17^. Most RT-qPCR experiments are performed by using a single reference gene for normalization. A study by Vandesompele *et al*. highlighted that *ACT1*, *GAPD1*, *RDN18*, and *RDN28* are the most frequently utilized single reference genes for normalization^21^. However, only a few studies have paid attention to the appropriate validation of candidate reference genes used in RT-qPCR based gene expression analysis^10,33–35^. In our study, the expression stability analysis of ten reference genes was performed to select the best internal controls for normalization. The expression levels of all the candidate genes were found to be differentially stable and suitable in fluconazole treated *C*. *tropicalis* isolates. To our knowledge, this is the first study to select and validate the appropriate reference genes for expression analysis in clinical isolates of *C. tropicalis*.

We used eight different methods including ∆∆CT, ∆CT, Pfaffl, hkgFinder, geNorm, NormFinder, BestKeeper, and web-based RefFinder for stability analysis. The most stable reference gene identified was variable among these eight methods, possibly due to the differences in their analytical algorithms^17,21,27–32^. Studies demonstrated that the expression stability of *RDN18*, *RDN28*, and *RDN5.8* was significantly higher compared to other reference genes in different experimental setup^17,36^. Similarly, stable expression of *RDN18* and *RDN28* was seen in the present study while the clear unsuitability of *RDN5.8* as an internal control was noted. The use of *RDN18* and *RDN28* has been recommended by several studies as an internal control for mRNA quantification^17,22–24^. However, since the transcript levels of *RDN18* and *RDN28* were very high (CT~5) and substantial sample dilution was required, it was difficult to correctly deduce the baseline values in expression analysis. As a result, *RDN18* and *RDN28* genes were excluded as reference genes despite their high stability.

Seven other reference genes with diverse functions were selected for further investigation in the present study. These genes could be categorized into the following classes: transcription-related genes (*EF1*), glycolytic enzymes (*GAPDH*, and *PGK1*), citric acid cycle enzyme (*SDHA*), cytoskeleton-related genes (*ACT1*, and *TUB1*), and Ubiquitin-conjugating enzyme (*UBC13*). Multiple studies had been performed using these genes as internal controls^17,22,23,37^. The present study demonstrated that *EF1* was one of the most stable genes, which is contradictory to the findings of Anita *et al*.^10^. *ACT1, TUB1*, and *SDHA* are also commonly utilized reference genes in stability analysis and target gene expression studies^10,17,34–36,38^. A study by Li *et al*. clearly demonstrated the unsuitability of *ACT1, TUB1*, and *SDHA* as reference genes for inducible expression analysis in *C. glabrata* cells treated with fluconazole^17^. In contrast, these genes were recommended as the most suitable and stable reference genes in *Microsporum canis* under various experimental conditions^10^. Such heterogeneous results may be due to the inherent biological characteristics of the different fungal isolates and/or variation in the test conditions. In the present study, the stability and suitability of *ACT1, SDHA*, and *EF1* were comparable making them all appropriate as reference genes for expression analysis in *C. tropicalis*. However, *TUB1* was found to be comparatively less stable. Additionally, *UBC13* was found to unsuitable in the present study, which is in contrast to the findings of Li *et al*.^17^. The *PGK1* and *GAPDH* genes play a significant role in the glycolytic pathway and variable degrees of expression stability have been reported by several studies^10,17,21,28,33–36,38,39^. This variation may be due to the difference in organisms selected for analysis and different experimental conditions utilized. *PGK1* and *GAPDH* had been shown to be co-regulated in a previous study^40^. However, such co-regulation was not evident in the present study and *GAPDH* was more stable compared to *PGK1*. The validation of *EF1, ACT1, GAPDH*, and *SDHA* as an internal control in azole resistance gene expression analysis was performed which confirmed either of these genes could be used as a potential reference gene. Thus, the arbitrary selection of a reference gene must be avoided and validation of internal controls across different experimental setup is essential.

Even though normalizing with a single reference gene is simple to use and widely accepted, some researchers have recommended the utilization of more than one reference gene for normalization^17,21,23,41^. This is the pragmatic approach to ensure correct normalization which is particularly essential when dealing with fine measurements. Vandesompele *et al*. documented that normalization using a single reference gene may lead to erroneous results and additional reference genes may be required^21^. Additionally, the stability analysis of multiple reference genes is not always possible, due to the less sample availability and significantly higher running cost. Furthermore, by using different combinations of multiple reference genes, the inter-experiment variability in result interpretation may increase. Using geNorm analysis, we found that a single reference gene was sufficient for providing accurate normalization and the combination of the reference gene is not required.

## Conclusion

In conclusion, the present study is the first to select and validate the reference genes in *C. tropicalis* for RT-qPCR based expression analysis. Our study highlights that, evaluation of the most appropriate internal control is an important prerequisite for RT-qPCR based expression analysis in different experimental models. The present study may also give preliminary knowledge for the assessment of candidate genes for expression studies in different *Candida* species and in diverse experimental conditions.

## Materials and Methods

### Isolates and growth conditions

The present study was conducted by following the Minimum Information for Publication of Quantitative Real-Time PCR Experiments (MIQE) guidelines^42^. Twenty fluconazole-resistant (16–256 mg/L) and 10 susceptible (0.5–1 mg/L) isolates of *C. tropicalis* from invasive candidiasis were used in this study. Of the 30 isolates collected from the patients, 26 from the blood, 2 from cerebrospinal fluid, and one each from pus and ascitic fluid (Supplementary Table S1). Informed consent was taken from each enrolled patient or a parent/ guardian if the patient is under 18 following our institute protocol. All the experiments used in this study was performed as per the guidelines and regulations approved by the institutional ethics committee of the Postgraduate Institute of Medical Education and Research (PGIMER), Chandigarh, India. All these isolates were also used in our previous studies and their details MICs are presented in Supplementary Table S1^43,44^. A total of 60 samples (30 fluconazole treated and 30 untreated controls) were used for stability analysis. The confirmation of identification was done by both PCR sequencing of the ITS region and by using MALDI-TOF MS (Bruker Daltonik, Bremen, Germany)^45,46^. *C. tropicalis* isolates were inoculated and grown in Yeast Extract-Peptone-Dextrose broth (YPD, HiMedia, India) at 30 °C with continuous shaking at 160 rpm. YPD broth in the presence (sub-inhibitory concentrations) and absence of fluconazole (Sigma-Aldrich, Germany) were supplemented with the freshly grown cells of *C. tropicalis* at a concentration of 1 × 10^6^ cells/mL and incubated up to 7 hours for RNA extraction^17^.

### Extraction of RNA and cDNA synthesis

Total RNA from *C. tropicalis* isolates was extracted at the logarithmic phase using TRIzol reagent (Invitrogen, USA) as per the manufacturer’s protocol. Both the quantity and quality of the extracted total RNA was analysed by determining the absorbance (A260/A280) by using a spectrophotometer (NanoDrop 2000/2000c, Thermo Scientific, USA). The RNA samples with 260/280 ratio of 1.85 to 2.06 were used in the present study. The integrity of the RNA samples was further examined by running in 1% denaturing agarose gel. RNase-free DNAse treatment (Qiagen, Germany) was given to each RNA preparation as per the manufacturer’s guidelines. High-Capacity cDNA Reverse Transcription Kit (Thermo Fisher Scientific, USA) was used for First-strand cDNA synthesis by using 1 µg of total RNA input in a 20 μL reaction volume. The PCR amplification was executed by using the standard protocol with the Eppendorf 5331 MasterCycler (Eppendorf, USA)^17^. During the synthesis of cDNA, two negative controls including no template control (NTC) without RNA input and no reverse transcriptase (NRT) controls were also analysed.

### Candidate gene selection and primer design

Ten candidate genes including *ACT1*, *EF1α*, *GAPDH*, *PGK1*, *RDN5.8*, *RDN18*, *RDN28*, *SDHA*, *TUB1*, and *UBC13* were examined for expression stability (Table 6). For the validation of reference genes 6 target genes [ABC transporter genes (*CDR1* and *CDR2*), Multi drug resistance gene (*MDR1*), Squalene epoxidase (*ERG1*), Δ^5,6^-desaturase (*ERG3*), Lanosterol C14α demethylase (*ERG11*)] related to azole resistance were also studied (Supplementary Table S2). The coding sequences of these candidate genes were obtained from NCBI (www.ncbi.nlm.nih.gov) and *Candida* Genome Database (www.candidagenome.org) (Table 6 and S2). Sequences of the selected genes were used to synthesize the primers by using the web-based Primer-Blast tool in NCBI and the quality of each primer was checked by using the online Sequence Manipulation Suite tool (www.bioinformatics.org) (Table 6 and S3). The binding efficiency of the synthesized primers was determined and the standardized optimum primer annealing temperature was 59 °C.

**Table 6:** List of candidate reference genes and details of primes used for stability analysis.

Gene symbol	Gene Name	Accession number	Sequence (5′->3′) forward and reverse	Amplicon length (bp)	Ta (ºC)
*ACT1*	β-actin	XM_002549283.1	CGTCGGTAGACCAAGACACC CCCAGTTGGAGACAATACCGT	137	59
*EF1*	Elongation factor 1α	XM_002547480.1	GGTCAAACCAGAGAACACGC TTCTTCAAATCTGTTTTTGTCCCA	111	59
*GAPDH*	Glyceraldehyde 3-phosphate dehydrogenase	XM_002551322.1	TTACGAAGAAATTTGTGCTGCT AGCATCAAAGACAGAGGAGTAAGA	130	59
*PGK1*	Phosphoglycerate kinase	XM_002548594.1	GCTGACGCTGTCGGTAAAG GCAGAAGCAACACAGGCA	116	59
*RDN5.8*	5.8S ribosomal RNA	AB437083.1	GAGCAATCCTACCGCCAGAG TGCGAGAACCAAGAGATCCG	113	59
*RDN18*	18S ribosomal RNA	M55527.1	GTGCTGGCGATGGTTCATTC CGTTTCTCAGGCTCCCTCTC	125	59
*RDN28*	28S ribosomal RNA	KY106836.1	GTGAAGCGGCAAAAGCTCAA CACCCTCTGTGACGTTCTGT	123	59
*SDHA*	Succinate dehydrogenase complex	XM_002549452.1	TTCGTAACCAAATAAGAAGTTCCGC GCTCATGTATTTGGCAGCGTTA	119	59
*TUB1*	α-tubulin	XM_002546417.1	TTGACTGGTGTCCAACTGGT CAGCAATAGCGGTAGTGTTAGA	126	59
*UBC13*	Ubiquitin-conjugating enzyme E2 13	XM_002550926.1	AGTATTCAAGCTTTGTTAGGTGCTC GAGTTTAGTCCATTCTTGAGCCAT	120	59

### RT-qPCR analysis

RT-qPCR amplification of reference genes and target genes was performed by using Light Cycler 480 (Roche, Switzerland). The 10 μL reaction mixture contained 5 μL of PowerUp SYBR Green Master Mix (Thermo Fisher Scientific, United States), 0.25 μL each primer (10 pmol), 1 μL cDNA and 3.5 μL nuclease-free ultrapure water. Amplification of cDNA templates was executed by employing the following conditions: starting denaturation cDNA templates at 95 °C for 1 min, then repetitions 45 cycles of denaturation at 94 °C for 10 seconds, annealing at 59 °C for 10 seconds, and extension at 72 °C for 10 seconds. Finally, the melting curve assessment was completed by applying the setup of denaturation at 95 °C for 5 seconds, annealing at 59 °C for 1 min and 97 °C for 15 seconds. The ‘CT’ or threshold cycle is the number of the cycle at which the reporter dye used in the PCR reaction crossed the software-designated threshold, which was automatically calculated by the Light Cycler 480 System Software (Roche, Switzerland) versions 1.5.

The calculated amplification efficiencies (E) for all the candidate genes used in this study were between 93.3–99.7% (Supplementary Table S5). The standard curves for the 16 genes were constructed by three fold serial dilutions and linear correlation coefficients (R^2^: 0.994–1.00) were observed (Supplementary Fig. S1). Each primer sets used in this study generated a single peak indicating the production of a single product (Supplementary Fig. S2). For each RT-qPCR experiment, two negative controls including one containing all the components without cDNA and another without primers were assessed simultaneously.

### Stability analysis of constitutively expressed genes

CT values from the RT-qPCR instrument were used for the stability analysis of reference genes to select the best genes for inducible expression analysis of pleiotropic target genes. The basal or constitutive expression level of reference genes was determined by comparing the fold changes with respect to a stable reference gene as a comparator by using ∆∆CT^27^, ΔCT^28^, Pfaffl^29^ approach. Five different software: hkgFinder^17^, geNorm^21^, NormFinder^30^, BestKeeper^31^, and RefFinder^32^ were used for the stability assessment of reference genes. The hkgFinder algorithm calculates the standard deviation (SD) of CT values obtained from both azole-untreated and treated cells of *C. tropicalis* and also calculates the fold changes among both phenotypes. The smallest SD of reference genes indicates the best reference gene. Another software, geNorm calculates both the stability value (M) and a pairwise variation (V). Both this analysis are used to examine the stability of any reference gene and to assess whether the combination of reference genes is required or not. The NormFinder software calculates the stability values depending on the intergroup and intragroup variability in the expression of different reference genes. The BestKeeper software determined the pairwise correlation to rule out the suitability of a reference gene with the BestKeeper Index, that is basically indicating the geometric mean (GM) of the acquired CT values. The BestKeeper algorithm commonly computes both the coefficients of variance CV(%CT) and standard deviation SD(±CT) for all the candidate reference genes. RefFinder is a web-based platform was utilized to assess and screen the candidate genes for stability ranking. It incorporates the most commonly used programs including BestKeeper, NormFinder, and geNorm to analyse and rank the reference genes. The recommended guidelines for each software package was followed by entering the raw RT-PCR data obtained as an output from the instrument, and the results were analysed accordingly^17,47^. For the validation of stable reference genes, the inducible overexpression expression of the resistance related genes was measured among the resistant isolates with respect to untreated control by utilizing the ∆∆CT method^27^.

## Electronic supplementary material

Supplementary material.
