# Supplementary material for: Selection and evaluation of appropriate reference genes for RT-qPCR based expression analysis in Candida tropicalis following azole treatment
Source: Sci Rep. 2020 Feb 6;10:1972. doi: 10.1038/s41598-020-58744-7 (PMC7004996; doi:10.1038/s41598-020-58744-7)

Selection and evaluation of appropriate reference genes for RT-qPCR based expression analysis in *Candida tropicalis* following azole treatment

Saikat Paul, Shreya Singh, Arunaloke Chakrabarti, Shivaprakash M. Rudramurthy, Anup K Ghosh*

Department of Medical Microbiology, Postgraduate Institute of Medical Education and Research (PGIMER), Chandigarh - 160012, India.

*** Corresponding author**.

Correspondence: Dr. Anup K Ghosh

Additional Professor

Department of Medical Microbiology,

Postgraduate Institute of Medical Education and Research (PGIMER),

Chandigarh 160012, India.

Email: anupkg3@gmail.com

Tel.: +91 172 2755156.

Fax: +91 172 2744401.

Supplementary Table S1. Details of resistant and susceptible isolates of *C. tropicalis*

SL No.	NCCPF ID	Source of isolates	Fluconazole MIC (mg/L)	Voriconazole MIC (mg/L)	Itraconazole MIC (mg/L)	Posaconazole MICs (mg/L)	NCBI Accession No.
1	420182	Blood	16	0.25	0.06	0.12	MF359893
2	420183	Cerebrospinal fluid	64	0.25	0.25	0.12	MF359894
3	420184	Blood	32	0.5	0.12	0.06	MF359895
4	420185	Blood	32	2	0.25	0.06	MF359896
5	420186	Blood	16	0.12	0.12	0.06	MF359897
6	420187	Blood	32	1	0.06	0.06	MF359898
7	420188	Blood	16	0.5	0.03	0.06	MF359899
8	420189	Blood	128	4	0.5	0.5	MF359900
9	420190	Blood	16	0.25	0.06	0.03	MF359901
10	420201	Blood	64	0.5	0.06	0.06	MF359902
11	420191	Blood	64	0.25	0.06	0.06	MF359903
12	420192	Blood	16	0.25	0.03	0.06	MF359904
13	420193	Blood	128	1	0.12	0.25	MF359905
14	420194	Blood	32	0.25	0.03	0.06	MF359906
15	420195	Blood	128	4	2	1	MF359907
16	420227	Pus	128	0.5	0.25	0.5	MK356074
17	420228	Blood	256	4	2	2	MK356075
18	420229	Blood	128	4	2	2	MK356076
19	420230	Blood	256	4	2	2	MK356077
20	420231	Cerebrospinal fluid	256	2	0.12	0.12	MK356078
21	420214	Blood	1	0.03	0.06	0.06	MF359925
22	420215	Blood	0.5	0.06	0.12	0.03	MF359926
23	420203	Blood	1	0.12	0.12	0.06	MF359914
24	420200	Blood	0.5	0.03	0.03	0.06	MF359912
25	420212	Blood	0.5	0.25	0.12	0.25	MF359923
26	420210	Blood	0.5	0.03	0.06	0.06	MF359921
27	420199	Blood	1	0.03	0.12	0.12	MF359911
28	420205	Ascitic fluid	1	0.25	0.12	0.06	MF359916
29	420204	Blood	0.5	0.06	0.12	0.03	MF359915
30	420198	Blood	0.5	0.12	0.06	0.03	MF359910

Supplementary Table S2. List of target genes and their accession numbers, used for validation of reference genes

SL. No.	Gene symbol	Gene Name	Accession No. of target sequences
1	*CDR1*	Candida drug resistance gene	XM_002548263.1
2	*CDR2*	Candida drug resistance gene	XM_002547994.1
3	*MDR1*	Multi drug resistance gene	XM_002548069.1
4	*ERG1*	Squalene epoxidase	XM_002551139.1
5	*ERG3*	$\Delta^{5,6}$-desaturase	XM_002550136.1
6	*ERG11*	Lanosterol C14α demethylase	XM_002550939.1

Supplementary Table S3. Sequences of primers for the expression analysis of target genes

SL. No.	Primer Name	Sequence (5'->3')	Product length (bp)	Annealing temperature (°C)
1	*CDR1*-F	TCGCCGTTTGCTGAAGAAGA	140	59
2	*CDR1*-R	GCAATCCCCAATTTCGATGGT		
3	*CDR2*-F	AAGGTGCAATCCAAAAGGGTG	101	59
4	*CDR2*-R	CTCAATATCACTGGGTGCTCCA		
5	*MDR1*-F	GCAGTTACCTCATCTGGAGCA	149	59
6	*MDR1*-R	GCACCAAACAATGGGAACACA		
7	*ERG1*-F	GAAAGAGTTCGTGGTGTTGCT	102	59
8	*ERG1*-R	AACGGTTGCTTCAACAGCAG		
9	*ERG3*-F	TTGGCAACTAGAGCCATTCCA	122	59
10	*ERG3*-R	AGTGCCTTATAACCACCAGTAGA		
11	*ERG11*-F	TTGCCATTCGGTGGTGGTAG	128	59
12	*ERG11*-R	ACATCTGGAACCTTATCACCGTT		

Supplementary Table S4. The pairwise variability analysis of all the constitutively expressed genes

GAPDH	0.058								
PGK 1	0.074	0.069							
RDN 5.8	0.079	0.102	0.091						
RDN 18	0.054	0.066	0.079	0.071					
RDN 28	0.054	0.073	0.087	0.070	0.031				
SDHA	0.057	0.074	0.086	0.087	0.058	0.053			
TUB1	0.066	0.074	0.075	0.108	0.087	0.086	0.078		
UBC 13	0.052	0.068	0.077	0.104	0.072	0.073	0.059	0.064	
EF 1	0.050	0.069	0.087	0.088	0.050	0.044	0.048	0.077	0.065
	ACT 1	***GAPDH***	***PGK 1***	***RDN 5.8***	***RDN 18***	***RDN 28***	***SDHA***	***TUB1***	***UBC 13***

Supplementary Figure S1. Standard curve of all the gene examined by using CT values and log cDNA dilutions

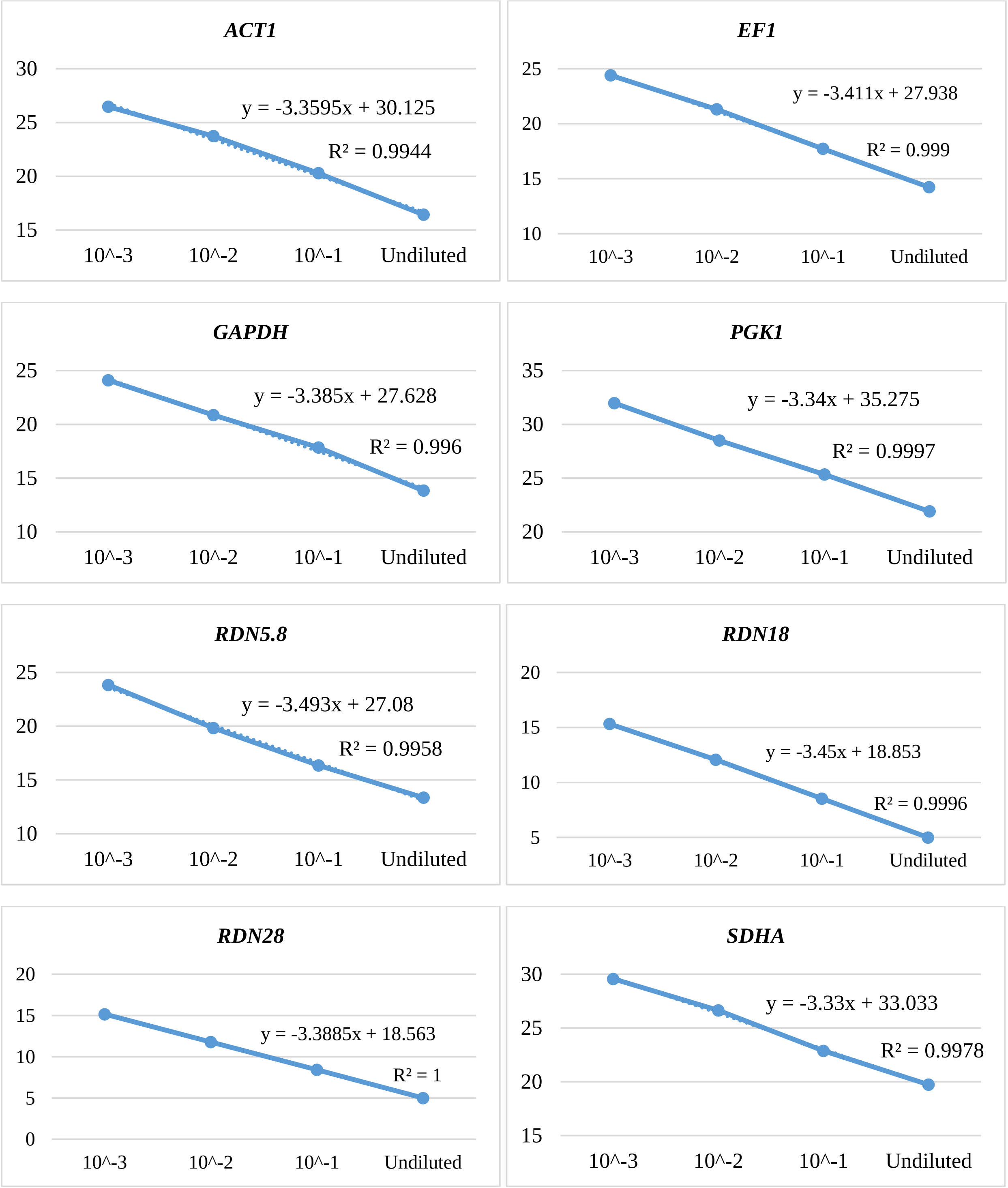

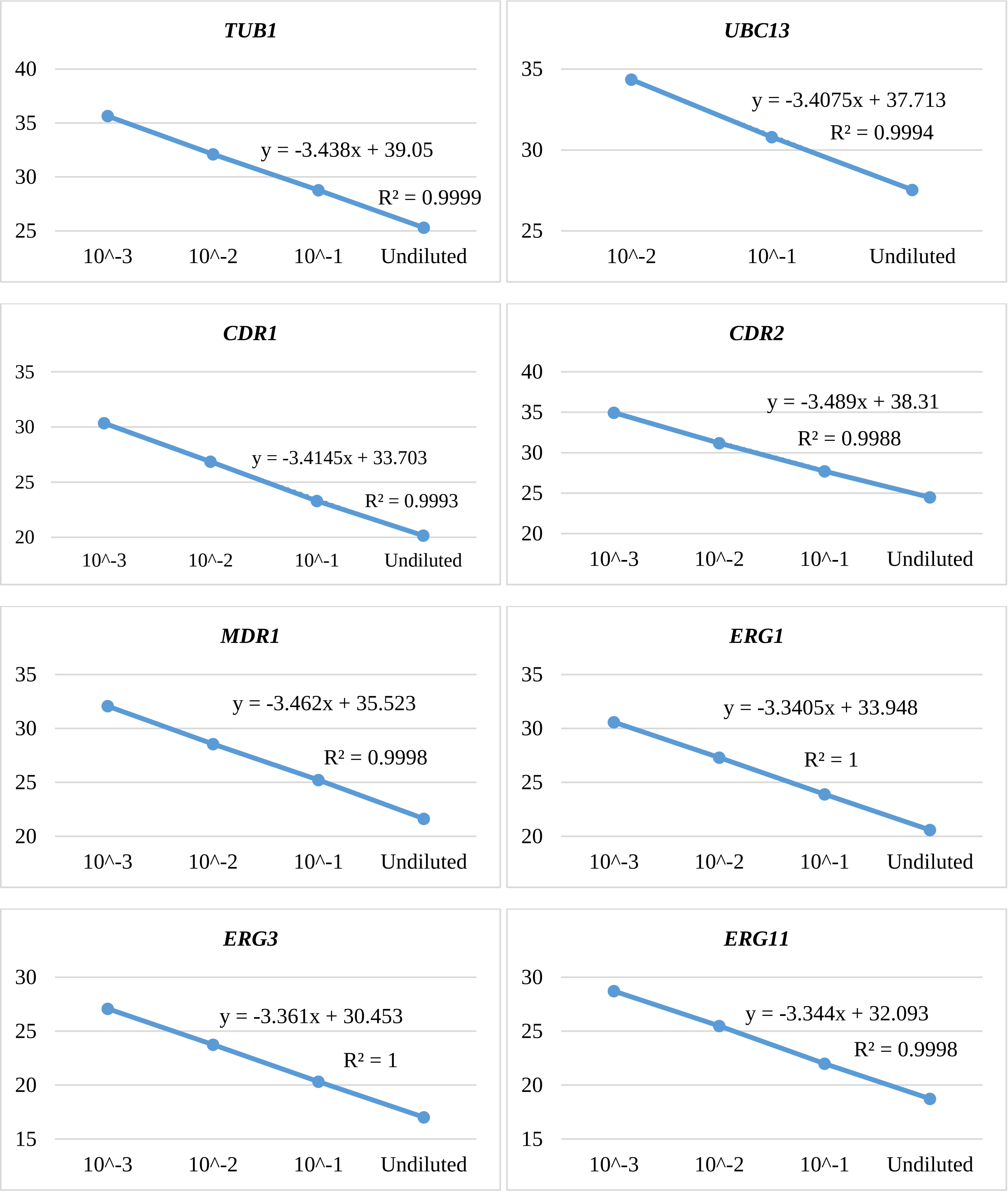
TUB1
40
35
30
25
y = -3.438x + 39.05
R² = 0.9999
10^-3
10^-2
10^-1
Undiluted
UBC13
35
30
25
y = -3.4075x + 37.713
R² = 0.9994
10^-2
10^-1
Undiluted
CDR1
35
30
25
20
y = -3.4145x + 33.703
R² = 0.9993
10^-3
10^-2
10^-1
Undiluted
CDR2
40
35
30
25
20
y = -3.489x + 38.31
R² = 0.9988
10^-3
10^-2
10^-1
Undiluted
MDR1
35
30
25
20
y = -3.462x + 35.523
R² = 0.9998
10^-3
10^-2
10^-1
Undiluted
ERG1
35
30
25
20
y = -3.3405x + 33.948
R² = 1
10^-3
10^-2
10^-1
Undiluted
ERG3
30
25
20
15
y = -3.361x + 30.453
R² = 1
10^-3
10^-2
10^-1
Undiluted
ERG11
30
25
20
15
y = -3.344x + 32.093
R² = 0.9998
10^-3
10^-2
10^-1
Undiluted

Supplementary Table S5. PCR amplification efficiency of the genes used in this study

	ACT1	***EF1***	***GAPDH***	***PGK1***	***RDN5.8***	***RDN18***	***RDN28***	***SDHA***	***TUB1***	***UBC13***	***CDR1***	***CDR2***	***MDR1***	***ERG1***	***ERG3***	***ERG11***
**Slope (m)**	-3.359	-3.466	-3.385	-3.340	-3.493	-3.450	-3.389	-3.330	-3.438	-3.408	-3.415	-3.489	-3.462	-3.341	-3.361	-3.344
**Amplification efficiency (E)**	1.985	1.943	1.974	1.993	1.933	1.949	1.973	1.997	1.954	1.965	1.963	1.935	1.945	1.992	1.984	1.991
**E-1**	0.985	0.943	0.974	0.993	0.933	0.949	0.973	0.997	0.954	0.965	0.963	0.935	0.945	0.992	0.984	0.991
**% Efficiency**	98.5	94.3	97.4	99.3	93.3	94.9	97.3	99.7	95.4	96.5	96.3	93.5	94.5	99.2	98.4	99.1

Supplementary Figure S2: Melting curves of 16 reference genes of *C. tropicalis* showing single peaks

ACT1

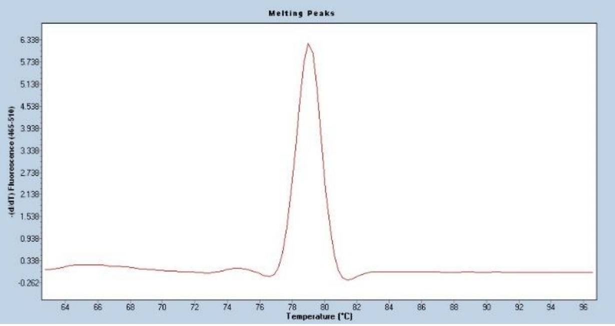

EF1

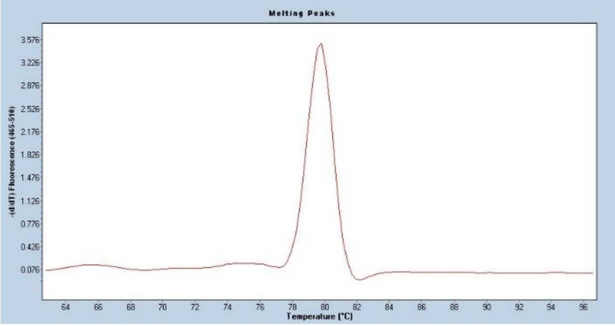

GAPDH

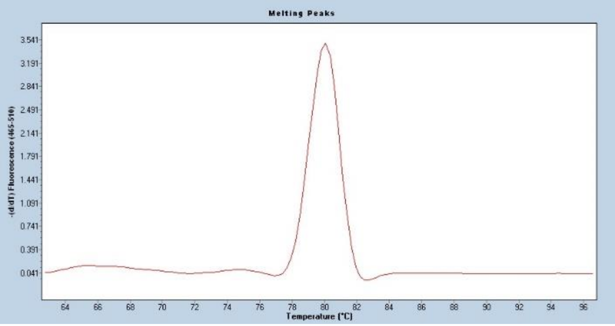

PGK1

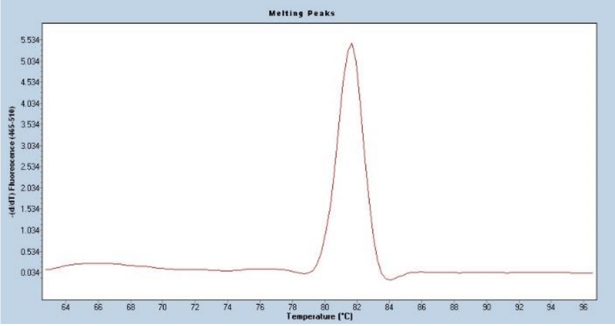

RDN5.8

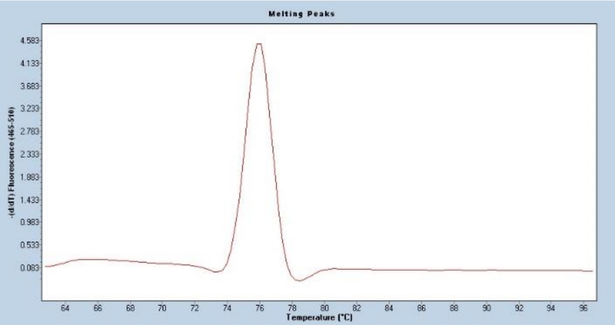

RDN18

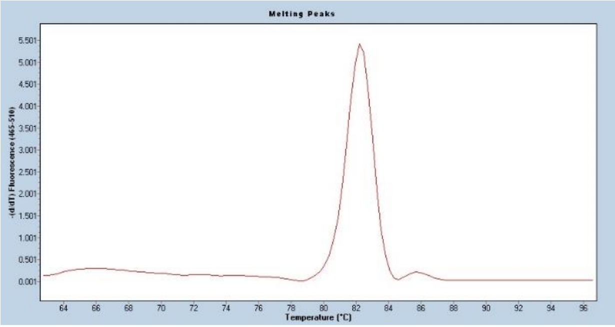

RDN28

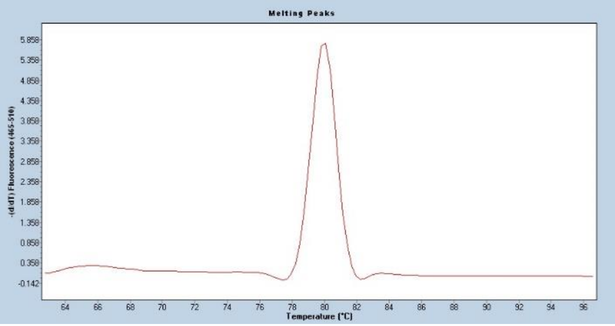

SDHA

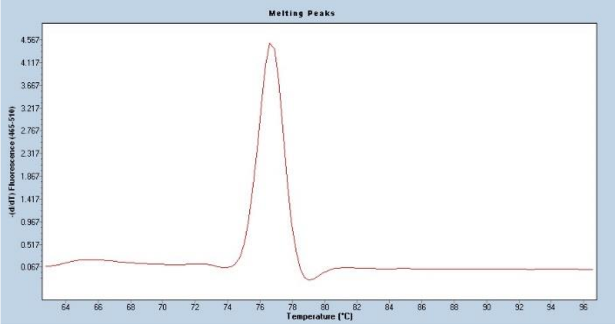

TUB1

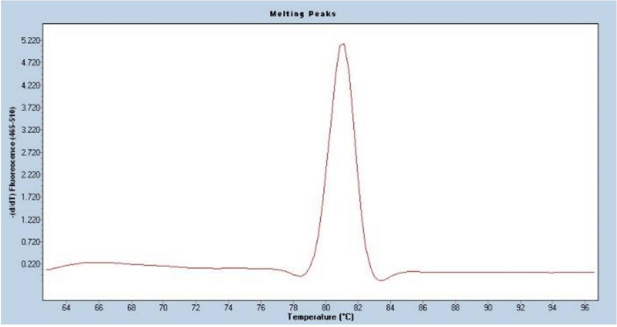

UBC13

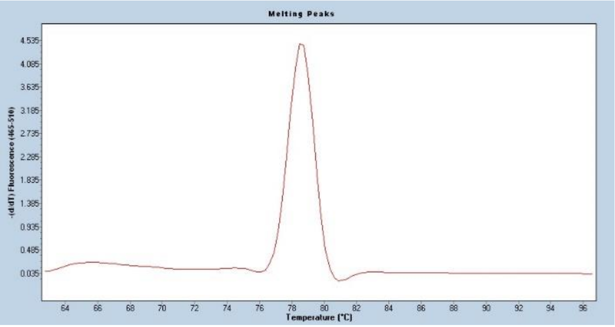

CDR1

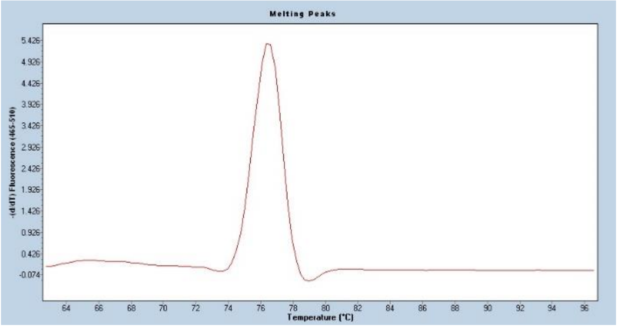

CDR2

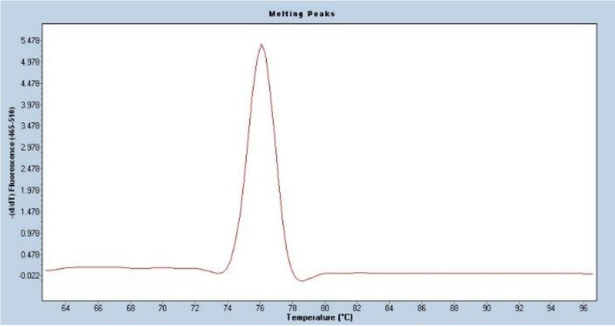

MDR1

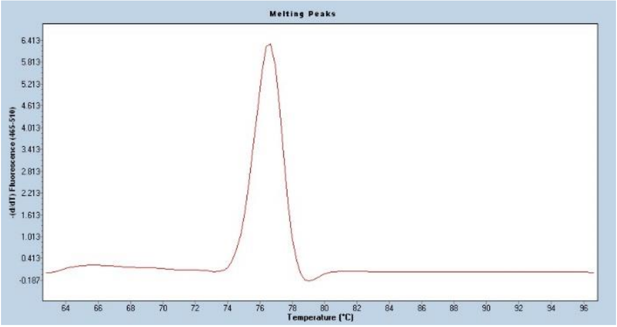

ERG1

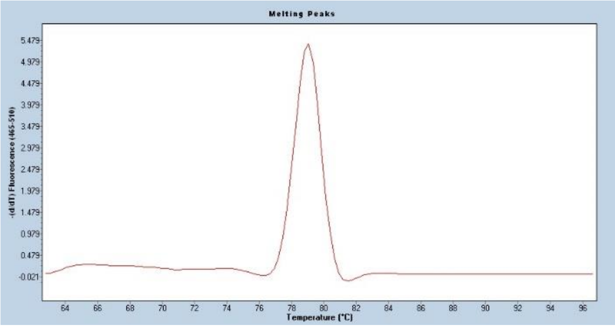

ERG3

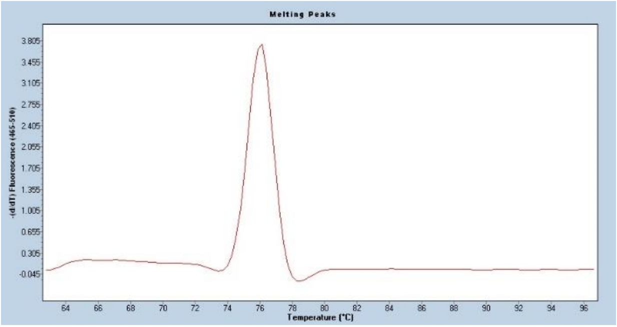

ERG11

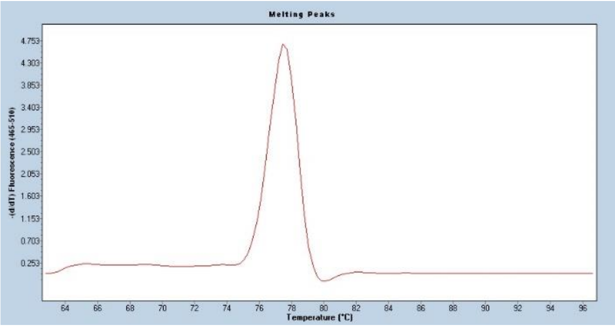

Supplement: Supplementary file 1 — Supplementary material. [file 41598_2020_58744_MOESM1_ESM.pdf]
